# Examining concepts related to critical illness and the management of critically ill patients: a protocol for a scoping review

**DOI:** 10.3310/nihropenres.14140.1

**Published:** 2026-01-14

**Authors:** Kirsty Jerrard, Brenda O'Neill, Judy M Bradley, Bronwen Connolly

**Affiliations:** 1 Wellcome-Wolfson Institute for Experimental Medicine, Queen's University of Belfast, Northern Ireland, BT9 7BL, UK; 2 School of Health Sciences, Ulster University Institute of Nursing and Health Research, Londonderry, Northern Ireland, BT48 7JL, UK; 3 Department of Physiotherapy, The University of Melbourne Melbourne Centre for Data Science, Melbourne, Victoria, Australia

**Keywords:** Critical illness, critical care, intensive care, concept analysis

## Abstract

**Background:**

Critically ill patients present with highly complex care needs that necessitate specialised, efficient collaboration across multidisciplinary teams. Effective communication and shared understanding of clinical concepts are imperative to delivering optimised care and enhancing patient outcomes. Concept analysis methodology offers a structured approach to clarifying complex clinical concepts to promote consistency in understanding and application. Gaining insight into concepts already described in this patient population, alongside identifying gaps, could support more collaborative understanding. This scoping review aims to systematically identify, synthesise, and map concepts underpinning the management of critically ill adult patients along the stages of the critical illness care pathway.

**Methods:**

We will search the databases MEDLINE ALL, EMBASE, CINAHL Complete, and Web of Science Core Collection from inception to date of search. Grey literature will be identified via ProQuest Dissertations & Theses Global. Reference lists of included studies will be screened to identify additional relevant literature. We will include studies using concept analysis methodology that explore, or define, concepts related to the management of critically ill adult patients at any stage of the care pathway. Screening and data extraction processes will be piloted and conducted independently and in duplicate. Extracted data will be analysed using descriptive statistics and narrative synthesis. Study characteristics will be summarised in tables. We will describe which concepts have been explored and how they have been defined, detail of the concept analysis method used, and in what populations and phases of the care pathway. Concepts will be mapped visually to illustrate their distribution across the pathway.

**Discussion:**

No reviews to date have summarised or mapped the concepts relating to the management of critically ill adult patients across the care pathway. Results of this review will inform clinical practice and help identify gaps for future research.

**Scoping review registration:**

This protocol is registered on OSF https://doi.org/10.17605/OSF.IO/Z63CW

## Plain English Summary

**Background:**

Patients who become critically ill may receive care in a range of settings, such as the emergency department, hospital ward, intensive care unit, rehabilitation services, and community after discharge home. Their treatment often needs input from many healthcare professionals working together. To make sure this care is safe and effective, clear communication and a shared understanding of medical concepts (ideas or ways of doing things) are essential. However, some of these concepts can be difficult to understand or may mean something different to individual healthcare professionals.

**Purpose:**

We want to understand which concepts have already been identified in relation to the care of critically ill adult patients in any setting.

**Methods:**

We will carry out an organised search of the research papers to find studies that explore these concepts. Two members of the research team will review the studies to decide which ones are relevant. For each relevant study, we will record key details in a spreadsheet, then summarise and describe the findings. We will also create a diagram to show how these concepts are used across different stages of critical illness care, from the beginning of treatment to recovery.

**Proposed benefits:**

The results of this review will help healthcare teams to develop a shared understanding about key concepts, improve communication, and work together more effectively. It will also highlight areas where further research is needed to improve care for critically ill adults. In addition, patients may benefit from knowing that these concepts have been explored, which could help them better understand their own critical illness journey.

## Background

Critically ill patients often present with life-threatening, complex, and multifaceted needs, requiring intensive treatment from multiple clinical specialities ^1^. Effective multidisciplinary team collaboration, both across and within specialities, is fundamental to delivering optimised, evidence-based, and high quality patient care ^2^. This is especially relevant in this population as the care pathway of critically ill patients can span a wide variety of settings including the emergency department, intensive care unit (ICU) and high dependency unit, general wards, rehabilitation settings, and ultimately the home/community environment following discharge. Care delivered both before and after ICU admission plays a crucial role in optimising outcomes for patients ^3, 4^. However, at each stage of the care pathway, patients may be managed by different teams, often with varying staffing levels and expertise, clinical priorities, professional cultures, and organisational pressures ^5^. These differences can lead to inconsistencies in care delivery, such as variations in treatment decisions, which may impact patient outcomes.

As the primary setting for the management of critically ill patients, the ICU is a distinct geographical and clinical setting, characterised by higher staff to patient ratios, specialised training in the management of critical illness, the use of high-risk and complex interventions, advanced physiological monitoring, and varying levels of care depending on the degree of organ support ^4^. For example, care may range from Level 1 (patients at risk of deterioration or requiring basic support) to Level 2 (those needing support for multiple organs or prolonged respiratory support), and Level 3 (patients requiring advanced respiratory or multi-organ support) ^6^. In such a complex and dynamic environment, a wide range of concepts ^7^ (principles or ideas) may be applied to guide clinical decision-making ^8^. However, when patients transition into or out of the ICU setting these concepts may be inconsistently understood or operationalised differently, resulting in fragmented patient care. A shared understanding of concepts is essential to support effective collaboration across teams, promote consistent use of terminology, and optimise patient outcomes; therefore, they require clear and agreed definitions. A lack of clarity and poor communication may lead to ambiguity, inconsistency in clinical practice, and an increased risk of patient harm ^5^.

One methodological approach used to clarify complex clinical concepts is concept analysis ^9^. This method has been applied in healthcare to define concepts such as clinical decision-making ^8, 10^, end-of-life care ^11^, and post-intensive care syndrome ^12^. Different frameworks to concept analysis have been described, including those by Walker and Avant ^13^, Rodgers ^14^, Schwartz-Barcott and Kim, Chinn and Kramer ^9^. These frameworks share some common elements, but also contrast with each other ^9^. For example, both Walker and Avant ^13^ and Rodgers ^14^ emphasise the importance of identifying the attributes of the concept, while Walker and Avant ^13^ and Chinn and Kramer ^9^ highlight the need to clearly choose the concept under analysis. However, Walker and Avant ^13^ employ a linear, stepwise process, whereas Rodger’s Evolutionary Method ^14^ adopts a cyclical model that emphasises iterative analysis, reflecting the dynamic nature of concepts ^9^.

Concept analysis typically involves several steps to clarify the meaning of a concept and explore its defining features ^9^, including selection of a concept of interest, determining the purpose of the analysis, identifying all the various uses of the concept (often through a literature review), and characterising its defining attributes. Walker and Avants approach also involves identifying antecedents (events or circumstances that must occur before the concept can arise) and consequences (outcomes or effects resulting from the presence of the concept), as well as developing exemplar case studies to illustrate the concept, clarify its boundaries and consider its practical application in context ^9^. For example, concept analysis of post-intensive care syndrome ^12^ using Walker and Avant’s framework clarified the multidimensional nature of the syndrome. By systematically reviewing relevant qualitative and quantitative literature, the analysis provided a comprehensive and holistic insight into the syndrome’s defining attributes and subsequently a theoretical basis to inform clinical practice, research, and future application. Regardless of the adopted approach, concept analysis provides an opportunity to enhance conceptual clarity and promote consistency in clinical practice.

Although various concepts relating to the care of critically ill patients have been explored through concept analysis in the literature ^11, 12, 15– 17^, there has been no systematic synthesis to date examining how these concepts map to the entire care pathway. Mapping which concepts have been explored in the literature could support shared understanding among multidisciplinary team members and facilitate knowledge translation across the critical illness care pathway, benefiting clinicians both within and beyond the ICU environment. This, in turn could inform clinical practice and guide future research.

Therefore, the aim of this review is to identify, synthesise, and map concepts related to critical illness and the management of critically ill adult patients across the stages of the care pathway.

### Rationale for a scoping review

A scoping review was chosen for this study as its methodology is well-suited to exploring and mapping broad, complex topics ^18^. This scoping review will examine diverse concepts, definitions, and the frameworks used to analyse these concepts in relation to the management of critically ill adult patients along the care pathway. A preliminary search of Open Science Framework registries and Figshare was conducted on 11 ^th^ July 2025 and no current or planned scoping reviews on this topic were identified. 

### Research question and objectives of the review

***Research question***

What concepts related to critical illness and the management of critically ill patients have been examined through concept analysis?

***Objectives***

1. To identify and describe the concepts that have been explored related to the management of critically ill adult patients.
2. To describe the concept analysis frameworks used to conduct these concept analyses.
3. To identify the populations, clinical contexts, and stages of the care pathway (e.g. pre-ICU, ICU, recovery) in which these concepts have been studied.
4. To visually map identified concepts to illustrate their distribution across the care pathway.

## Protocol

### Methodology and registration

The protocol was developed in accordance with updated JBI recommendations ^18^ and the PRISMA Extension for Scoping Reviews (PRISMA-ScR) Checklist ^19^. It was reviewed and revised by the study team (KJ, BO’N, JB, BC) before being finalised. The final protocol was registered prospectively on the Open Science Framework (OSF) registries ( https://doi.org/10.17605/OSF.IO/Z63CW) ^20^ on 31 ^st^ July 2025. This scoping review forms part of a process evaluation of the NIHR Health Technology Assessment Programme-funded MARCH trial in which concept analysis will be employed as one methodological and analytical approach (MARCH; Mucoactive drugs for acute respiratory failure: A 2×2 factorial, randomised, controlled, open-label, Phase 3, pragmatic, clinical and cost effectiveness trial with internal pilot, https://www.fundingawards.nihr.ac.uk/award/NIHR130454).

### Patient and Public Involvement

Patients and members of the public were first involved in this research during the planning phase, through consultation with the MARCH trial’s Patient and Family Advisory Group (PFAG) The aim, purpose, and proposed methods of this review were discussed with the PFAG, who expressed strong support and highlighted the novelty and relevance of the study.

PFAG members were keen to understand who defines the concepts used in clinical care, what sources of literature are used to support these definitions, and which stakeholders are involved in shaping them. Their lived experience informed the development of the research questions, particularly around improving communication and understanding through clearer, more standardised terminology used by healthcare professionals across different care settings (which reflected their real-world experience).

PFAG members also contributed to the design and conduct of the review, by raising important context-specific considerations. These included geographical variation in clinical practice, differences in service provision, and resource availability. They recommended that the review be transparent about the applicability and transferability of its findings across different healthcare jurisdictions and settings. This feedback will be reflected in data analysis, interpretation, and discussion.

One member provided feedback on the plain English summary, noting it was clear and emphasising that this is a vital piece of research for healthcare professionals, patients and their loved ones. PFAG members were also interested in understanding how the review could benefit patients and how its findings could be shared with both healthcare professionals and patients at different stages of the critical illness journey. The group agreed to share their perspectives on the review findings, and to co-develop strategies for effective dissemination.

### Eligibility criteria

Studies will be selected according to the Population-Concept-Context framework ^18^.

***Population***

We will include studies relating to adult patients, aged 16 years and over, with critical illness (as defined by the original study authors), regardless of diagnosis or underlying condition.

***Concept***

We will include studies that explicitly use any concept analysis method to explore, define, or clarify concepts relating to the management of critically ill adult patients.

***Context***

We will include studies conducted at any stage of the patient’s critical illness care pathway, including but not limited to:

- Pre-ICU care (e.g. emergency department, ward)
- ICU and high dependency unit (HDU)
- Post-ICU care (e.g. ward, rehabilitation unit, follow-up clinic)

***Types of studies***

We will include any study types that align with the research question. To maximise inclusivity, no language restrictions will be applied. Non-English publications will be translated where local resources permit.

### Information sources and search strategy

To identify potentially relevant literature, we will search the following electronic databases for full text published literature: MEDLINE ALL, EMBASE, CINAHL Complete, and Web of Science Core Collection from inception until date of search. To capture grey literature, we will search ProQuest Dissertations & Theses Global using a combination of key terms related to the population, concept, and context. To assess the relevance of grey literature, 10% of the most relevant search results will be screened by one reviewer (KJ). Following the initial screening, the study team will determine whether further review of the remaining grey literature is warranted. Only full-text sources will be included.

Where full texts are unavailable, the reviewer (KJ) will contact the primary or corresponding author to confirm publication status. Finally, the reference lists of included studies and the research team’s personal libraries will be reviewed to identify any additional relevant studies.

The search strategy was initially drafted by one of the reviewers (KJ) and subsequently refined in collaboration with an experienced health science librarian to identify suitable MeSH terms and keywords. Final search strategies for MEDLINE ALL and ProQuest Dissertations & Theses Global are provided as additional project files associated with the OSF registration ^20, 21^. The search strategy will be adapted as needed for each database to ensure comprehensive retrieval of relevant literature.

### Screening, selection, and data extraction procedure

Search results will be collated and uploaded into reference software management programme EndNote 21 (Clarivate Analytics, PA, USA) by one reviewer (KJ) to allow for removal of duplicates and non-relevant results. Remaining search results will be imported into a systematic review programme, Covidence (Veritas Health Innovation, Melbourne, Australia), for screening. To ensure rigour, the screening process will be piloted by two reviewers (KJ and one other); a random sample of up to 25 titles and abstracts will be screened using the eligibility criteria, any discrepancies will be discussed. Following completion of the pilot, all titles and abstracts will be screened independently by two reviewers (KJ and one other). Potentially relevant sources will be retrieved in full along with any associated supplementary material and imported into Covidence. The full text of selected citations will be assessed in detail for inclusion independently by two reviewers (KJ and one other). Reasons for excluding sources of evidence at full text stage will be recorded and reported. The results of the search and the study inclusion process will be reported in a PRISMA flow diagram.

Data will be extracted into a pre-defined Microsoft Excel database, initially developed by one reviewer (KJ) and finalised in collaboration with the study team (KJ, BO’N, JB, BC). The data extracted will focus on:

1. Individual study characteristics (e.g. author, year, country of conduct, setting)
2. Population characteristics (e.g. whether the concept is related to a specific disease or condition)
3. Concept characteristics (e.g. concept name, description, clinical application, detail of method)
4. Context characteristics (e.g. the stage of the patient’s critical illness care pathway– pre-ICU, ICU, or recovery)

The data extraction template will be piloted by two reviewers (KJ and one other) to confirm all relevant data are captured in a consistent manner and ensure consistency in application. An iterative approach will also be used to refine the template as appropriate, should new insights regarding data extraction emerge during the review. Any modifications made will be detailed in the final report. Data from each eligible study will be extracted independently by two reviewers (KJ and one other) and then cross-checked for completeness. Data will be stored on a university (Queen’s University Belfast) server.

Any disagreements in screening and data extraction between reviewers will be resolved via discussion, with arbitration by a third reviewer, if necessary, where consensus cannot be achieved.

### Risk of bias assessment

In keeping with established scoping review methodology, a formal assessment of risk of bias for the included studies will not be conducted. Risk of bias assessment would not influence data extraction, analysis, or synthesis.

### Data analysis and synthesis

Extracted data will be analysed using descriptive statistics and narrative synthesis. Study characteristics will be summarised and presented in tabular form. We plan to visually map the identified concepts in diagrammatic form, aligned with the phases of the critical illness care pathway (e.g. pre-ICU, ICU, or recovery), to illustrate how these concepts are distributed across the pathway. Additionally, we will identify whether each concept identified is broadly applicable to all critically ill adult patients or specific to a particular subgroup of disease or condition.

## Discussion

This scoping review will provide a comprehensive overview of concepts related to critical illness and the management of critically ill adult patients that have been examined using concept analysis methodology. Mapping these concepts across different stages of the critical illness care pathway and identifying their clinical and population specific applications, this review will offer valuable insights into current conceptual understandings in this area. It is anticipated that the findings will help inform clinical practice, guide future research, and highlight potential areas in existing literature which would benefit from further exploration. Importantly, the review may also benefit patients and their families by supporting clearer communication and improving their understanding of key concepts used throughout the critical illness journey, helping them feel more informed and engaged in their care.

The review will adhere to rigorous JBI methodology ^18^ and will be reported in accordance with PRISMA-ScR guidelines ^19^, ensuring a systematic and transparent approach. To enhance transparency and mitigate the risk of reporting bias, the protocol has been prospectively registered on OSF ^20^. Search strategies were developed in collaboration with expert input to maximise the identification of potentially relevant literature. These strategies encompass a broad range of databases as well as unpublished and grey literature. To promote inclusivity, we will include studies written in any language. Screening and data extraction will be piloted and conducted independently by two reviewers to ensure consistency and reduce bias. Finally, the research team comprises individuals with experience in both clinical practice and research methodology, which will ensure robust data identification and interpretation. One potential limitation of the review is the absence of a formal quality appraisal of the included studies, though this falls outside the remit of a scoping review. Furthermore, given the heterogeneity of critically ill patients and the different management needed for subsequent treatment, this may result in diverse concepts identified in the literature. Our planned approach to synthesis should offer some mitigation to this by considering the generalisability versus specificity of these concepts.

### Dissemination

Following completion of the synthesis, the results of this review will be submitted for publication in a peer-reviewed journal. We will also engage with the MARCH PFAG to discuss how best to share the results with patients and the public, ensuring the findings are accessible and meaningful across different stages of the critical illness journey.

### Ethics approval and consent to participate

As this study is a scoping review of published literature, it does not involve the collection of new data from human participants and therefore does not require ethical approval.

## List of abbreviations

PFAG; Patient and Family Advisory Group

PRISMA-ScR; Preferred Reporting Items for Systematic Reviews and Meta-Analyses Protocols Extension for Scoping Reviews

ICU; Intensive Care Unit

OSF; Open Science Framework

## Data Availability

No underlying data are associated with this article as it describes a study protocol rather than reporting study findings. Additional files supporting this article can be accessed via the Open Science Framework. The registered protocol is available at https://doi.org/10.17605/OSF.IO/Z63CW ^20^ Project files can be accessed at https://doi.org/10.17605/OSF.IO/BQFK5 ^21^ This project contains the following files: Additional file 1: PRISMA-ScR Checklist Description of data: The completed PRISMA-ScR Checklist, which outlines how each item was addressed in the scoping review. Additional file 2: Search strategy Description of data: Tables presenting the comprehensive search strategy used to identify relevant literature for this review. Includes detailed search strings applied to both published literature databases (MEDLINE ALL) and grey literature sources (ProQuest Dissertations & Theses Global), structured using the Population, Concept, and Context (PCC) framework. Additional file 3: Data extraction form Description of data: Predefined Microsoft Excel-style data extraction template used in the scoping review. Outlines key fields for capturing study characteristics, population details, conceptual focus, and contextual information from included studies. The protocol was developed in accordance with the PRISMA Extension for Scoping Reviews (PRISMA-ScR) Checklist, available at https://doi.org/10.17605/OSF.IO/BQFK5 ^21^. Data are available under the terms of the Creative Commons Attribution 4.0 International license (CC-BY 4.0).
