# Supplementary material for: Examining concepts related to critical illness and the management of critically ill patients: a protocol for a scoping review
Source: NIHR Open Res. 2026 Jul 30;6:8. Originally published 2026 Jan 14. [Version 3] doi: 10.3310/nihropenres.14140.3 (PMC12972711; doi:10.3310/nihropenres.14140.3)
Supplement: Peer review history

# Reviewer report: Reviewer response for version 3

**DOI:** 10.3310/nihropenres.15720.r41369

**Published:** 2026-08-03

**Authors:** Lisa Salisbury

**Affiliations:** 1 Queen Margaret University, Musselburgh, Scotland, UK

No further comments.

Is the study design appropriate for the research question?

Yes

Is the rationale for, and objectives of, the study clearly described?

Yes

Are sufficient details of the methods provided to allow replication by others?

Yes

Are the datasets clearly presented in a useable and accessible format?

Not applicable

Reviewer Expertise:

Evaluation of rehabilitation interventions after critical illness; Experiences of individuals after critical illness.

I confirm that I have read this submission and believe that I have an appropriate level of expertise to confirm that it is of an acceptable scientific standard.

# Reviewer report: Reviewer response for version 2

**DOI:** 10.3310/nihropenres.15546.r40063

**Published:** 2026-03-19

**Authors:** Porkodi Arjunan

**Affiliations:** 1 King Faisal University, Al Hofuf, Saudi Arabia

Thanks for submiting the revised version based on the comments. 

 The authors are advised to:

- Clarify whether the care pathway is intended to be generic across conditions or tailored to specific disease processes.
- Ensure alignment between the defined concept and the stages of care (pre-ICU, ICU, recovery).

Improving this clarity will enhance the rigor, consistency, and applicability of the review

Is the study design appropriate for the research question?

Yes

Is the rationale for, and objectives of, the study clearly described?

Partly

Are sufficient details of the methods provided to allow replication by others?

Yes

Are the datasets clearly presented in a useable and accessible format?

Not applicable

Reviewer Expertise:

critical care nursing, cardiac rehabilitation, medical surgical nursing

I confirm that I have read this submission and believe that I have an appropriate level of expertise to confirm that it is of an acceptable scientific standard.

# Reviewer report: Reviewer response for version 2

**DOI:** 10.3310/nihropenres.15546.r40064

**Published:** 2026-03-19

**Authors:** Lisa Salisbury

**Affiliations:** 1 Queen Margaret University, Musselburgh, Scotland, UK

No further comments

Is the study design appropriate for the research question?

Yes

Is the rationale for, and objectives of, the study clearly described?

Yes

Are sufficient details of the methods provided to allow replication by others?

Yes

Are the datasets clearly presented in a useable and accessible format?

Not applicable

Reviewer Expertise:

Evaluation of rehabilitation interventions after critical illness; Experiences of individuals after critical illness.

I confirm that I have read this submission and believe that I have an appropriate level of expertise to confirm that it is of an acceptable scientific standard.

# Reviewer report: Reviewer response for version 1

**DOI:** 10.3310/nihropenres.15386.r39544

**Published:** 2026-03-09

**Authors:** Heather K O'Grady

**Affiliations:** 1 Niagara Health Knowledge Institute, Niagara Health System (Ringgold ID: 27363), Saint Catharines, Ontario, Canada

Jerrard and colleagues report a protocol for a scoping review of concept analyses related to critical care and the management of critical illness. The authors provide a clear, detailed description of the proposed methods.

 Thank you very much for the opportunity to review this manuscript! This work addresses an important question in the multidisciplinary field of critical care. Particularly, the authors are to be congratulated for a comprehensive review protocol, with the inclusion of patient and public involvement. This protocol manuscript is well written and transparently reported.

 In general, I agree with the other peer reviewers that the scope of this review could use some clarification, particularly in the title and the background. While I feel the methods were clearly reported, the background was vague. For the title, I might suggest something a little closer to your research question (e.g., A Scoping Review of Concept Analyses Related to Critical Illness). It wasn’t until I got to this research question that I truly understood the design of this project. In the background, perhaps providing some upfront examples of the concepts you are considering or that might be included could be helpful. For example, are you only interested in concepts related to the biopsychosocial care for critically ill patients? The references you cite (11, 12, 15-17) seem to suggest this. Or would you also include concepts related to other elements of critical care such as family experiences, communication and decision making, care processes etc.? Additional explanation in the background and in your inclusion criteria would enhance clarity for readers.

 Below, I will have included some minor comments for consideration.

1. Patient and Public Involvement – I suggest revising the following sentence “PFAG members also contributed to the design and conduct of the review, by raising important context-specific considerations” to drop “and conduct”. It is a bit confusing for the reader as it sounds like the PFAG was actually involved in conducting the review, where if I understand correctly they were involved in informing the design and protocol.
2. Screening and Data Extraction – some methods for screening and data extraction are unclear. In the abstract, the authors state that screening and data extraction processes will be conducted “independently and in duplicate”. However, in the methods, it seems that review and data extraction are occurring independently by one of two reviewers. It would be helpful to clarify what, if any processes will be conducted in duplicate (i.e., two reviewers looking at the same citation or data) versus what is being done by one of two reviewers.
3. Disagreements – following up on my previous comment, if it is only the piloting of screening and data extraction that are being done in duplicate, it is unclear why you would have disagreements that need to be resolved (i.e., if only one reviewer is looking at each citation, then there shouldn’t be any disagreements). Additional clarification would be helpful!
4. Grey Literature – the authors indicate that they are including grey literature in the review. However, it appears that the database selected only reflects one type of grey literature (theses/dissertations). If this is the case, I suggest revising to more clearly reflect that you are including concept analyses reported in published manuscripts or in theses/dissertations.
5. Discussion – the authors state that “the findings will help inform clinical practice, guide future research and highlight potential areas in existing literature which would benefit from further exploration.” Can the authors briefly elaborate on *how* they expect findings to inform clinical practice and guide future research? I do not disagree with this point, but by explicitly addressing this in the discussion, the impact of this important work will be more readily understood.
6. Dissemination – these are amazing considerations! I look forward to seeing your results and any dissemination tools you create with your PFAG.

Is the study design appropriate for the research question?

Yes

Is the rationale for, and objectives of, the study clearly described?

Yes

Are sufficient details of the methods provided to allow replication by others?

Partly

Are the datasets clearly presented in a useable and accessible format?

Not applicable

Reviewer Expertise:

Literature reviews, knowledge translation, critical care rehabilitation

I confirm that I have read this submission and believe that I have an appropriate level of expertise to confirm that it is of an acceptable scientific standard, however I have significant reservations, as outlined above.

# Response

**Published:** 2026-07-23

**Authors:** Kirsty Jerrard

**Affiliations:** Queen's University Belfast Wellcome-Wolfson Institute for Experimental Medicine, Belfast, Northern Ireland, UK

Thank you for taking the time to review our protocol and for your positive comments around the  comprehensiveness and transparent reporting of our protocol, and recognition of  the value of the patient and public involvement incorporated within the study. We appreciate your constructive suggestions and have responded to each comment in detail below.

1. Comment:

 I agree with the other peer reviewers that the scope of this review could use some clarification, particularly in the title and the background. For the title, I might suggest something a little closer to your research question (e.g., A Scoping Review of Concept Analyses Related to Critical Illness). It wasn’t until I got to this research question that I truly understood the design of this project.

Response:

 We agree that the scope of the review required further clarification. In Version 2 of the protocol, we refined the aim and objectives to provide a more explicit description of the review's scope, including its focus on concepts related to critical illness and the management of critically ill adult patients across the critical illness care pathway. Specifically, the aim was revised to state: *"To identify, synthesise, and map the concepts that underpin critical illness and the management of critically ill adult patients across the stages of the care pathway."*

 These revisions were intended to improve readers' understanding of the review's purpose and design earlier within the manuscript. While no specific amendments were made to the Background section in response to this comment, the revisions to the aim and objectives were intended to provide greater clarity regarding the review scope and purpose.

 We appreciate your suggestion regarding the title. Following consideration, we have retained the current title in the context of this protocol as we believe it reflects the breadth of the review and its focus on concepts related to both critical illness and its management. However, we acknowledge the value of your suggestion and will revisit the title when preparing the final review manuscript for publication to ensure maximum clarity.

2. Comment: In the background, perhaps providing some upfront examples of the concepts you are considering or that might be included could be helpful. For example, are you only interested in concepts related to the biopsychosocial care for critically ill patients? The references you cite (11, 12, 15-17) seem to suggest this. Or would you also include concepts related to other elements of critical care such as family experiences, communication and decision making, care processes etc.? Additional explanation in the background and in your inclusion criteria would enhance clarity for readers.

Response:

 We agree that providing clearer examples of the types of concepts eligible for inclusion would improve readers' understanding of the review scope. In Version 2, we clarified that the review is not restricted to concepts related to the biopsychosocial care of critically ill patients. Rather, our intention is to map concepts identified across the critical illness care pathway and across multiple stakeholder groups, including patients, family members, healthcare professionals, and organisational or operational contexts. To reflect this broader scope, we revised the review objectives to state: *“To identify the populations, clinical contexts, and stages of the care pathway (e.g. pre-ICU, ICU, recovery) in which these concepts have been studied, including concepts relating to patient-level management, family involvement, healthcare professional practice, and organisational or operational processes.”*

 While the examples cited in the background (references 11, 12, 15-17) are predominantly patient-focused, these references were intended as illustrative examples rather than an exhaustive representation of the concepts eligible for inclusion. The revised objective was added to make the intended breadth of the review clearer.

3. Comment: Patient and Public Involvement – I suggest revising the following sentence “PFAG members also contributed to the design and conduct of the review, by raising important context-specific considerations” to drop “and conduct”. It is a bit confusing for the reader as it sounds like the PFAG was actually involved in conducting the review, where if I understand correctly they were involved in informing the design and protocol.

Response:

 We agree that the original wording could have implied that PFAG members were involved in conducting the review itself. To avoid this misunderstanding, the sentence was revised in Version 2 to state that PFAG members *“contributed to the design of the review by raising important context-specific considerations.”* This wording more accurately reflects their role in informing the development of the protocol and review design, rather than the conduct of the review.

4. Comment: Screening and Data Extraction – some methods for screening and data extraction are unclear. In the abstract, the authors state that screening and data extraction processes will be conducted “independently and in duplicate”. However, in the methods, it seems that review and data extraction are occurring independently by one of two reviewers. It would be helpful to clarify what, if any processes will be conducted in duplicate (i.e., two reviewers looking at the same citation or data) versus what is being done by one of two reviewers.

 Disagreements – following up on my previous comment, if it is only the piloting of screening and data extraction that are being done in duplicate, it is unclear why you would have disagreements that need to be resolved (i.e., if only one reviewer is looking at each citation, then there shouldn’t be any disagreements). Additional clarification would be helpful!

Response:

 Thank you for highlighting this point. We would like to clarify that all stages of screening (title/abstract and full-text screening) and all data extraction will be conducted independently and in duplicate by two reviewers. To improve clarity, we have revised the wording throughout the Methods section to explicitly state screening and data extraction will be undertaken independently and in duplicate by two reviewers. The pilot exercises are intended to ensure consistent application of the eligibility criteria and data extraction template prior to commencing the full review; however, duplicate review is not limited to the piloting stage. As a result, disagreements may arise at any stage of screening or data extraction and will be resolved through discussion, with arbitration by a third reviewer if consensus cannot be reached. We appreciate the opportunity to clarify this aspect of the methodology.

5. Comment: Grey Literature – the authors indicate that they are including grey literature in the review. However, it appears that the database selected only reflects one type of grey literature (theses/dissertations). If this is the case, I suggest revising to more clearly reflect that you are including concept analyses reported in published manuscripts or in theses/dissertations.

Response:

 We acknowledge that ProQuest Dissertations & Theses Global represents a specific source of grey literature rather than the full range of grey literature sources available. Our intention was to identify unpublished literature, specifically theses and dissertations, alongside concept analyses reported in the peer-reviewed literature, as these are considered the most likely unpublished sources relevant to the review question. To improve clarity, we have revised the wording in both the Abstract and Methods sections to reflect this scope. We have retained the current search strategy but appreciate the distinction you have highlighted and will acknowledge this as a potential limitation in the final review report.

6. Comment: Discussion – the authors state that “the findings will help inform clinical practice, guide future research and highlight potential areas in existing literature which would benefit from further exploration.” Can the authors briefly elaborate on *how* they expect findings to inform clinical practice and guide future research? I do not disagree with this point, but by explicitly addressing this in the discussion, the impact of this important work will be more readily understood.

Response:

 Thank you for this comment. We agree that it is important to consider how the findings of this review may inform clinical practice and future research. In response, we have expanded the Discussion section to provide additional detail regarding the anticipated impact of the review findings. Specifically, we have clarified that, by identifying and mapping concepts related to critical illness and the management of critically ill patients, the review may improve conceptual clarity and facilitate knowledge translation among multidisciplinary team members. This may support greater consistency in the interpretation and application of concepts across different stages of the critical illness care pathway.

 We have also highlighted that the review will identify concepts that have already been explored through concept analysis, as well as areas where conceptual clarification may still be required. In doing so, the review may help researchers identify gaps in the literature and prioritise future concept development work. We appreciate the opportunity to further clarify the anticipated contribution and impact of the review.

# Reviewer report: Reviewer response for version 1

**DOI:** 10.3310/nihropenres.15386.r39548

**Published:** 2026-03-04

**Authors:** Porkodi Arjunan

**Affiliations:** 1 King Faisal University, Al Hofuf, Saudi Arabia

The aim of the review requires further clarification. The phrase *“concepts related to critical illness”* may include a broad range of aspects. It is therefore unclear whether the review focuses specifically on the management of critically ill adult patients or on critical illness concepts. The authors are encouraged to clearly specify the scope of the review and refine the aim accordingly.

 The authors are encouraged to clarify whether the review intends to comprehensively address management across all three phases or focus on specific aspects within each stage. Narrowing or clearly defining the scope of management within these phases would improve the clarity and feasibility of the objective.

 The population is described as adults; however, the inclusion criteria specify participants aged 16 years and above.  The authors should provide a clear justification for including individuals aged 16–17 years in the adult population. 

 The section describing the types of studies should clearly specify the time frame for the included studies. The authors are encouraged to mention the range of publication years considered in the review. 

Is the study design appropriate for the research question?

Yes

Is the rationale for, and objectives of, the study clearly described?

Partly

Are sufficient details of the methods provided to allow replication by others?

Yes

Are the datasets clearly presented in a useable and accessible format?

Not applicable

Reviewer Expertise:

critical care nursing, cardiac rehabilitation, medical surgical nursing

I confirm that I have read this submission and believe that I have an appropriate level of expertise to confirm that it is of an acceptable scientific standard, however I have significant reservations, as outlined above.

# Response

**Published:** 2026-03-06

**Authors:** Kirsty Jerrard

**Affiliations:** Queen's University Belfast Wellcome-Wolfson Institute for Experimental Medicine, Belfast, Northern Ireland, UK

We thank the reviewer for taking the time to review our manuscript and for their helpful feedback. Our responses to the comments are outlined below.

1. Comment:

 The aim of the review requires further clarification. The phrase “concepts related to critical illness” may include a broad range of aspects. It is therefore unclear whether the review focuses specifically on the management of critically ill adult patients or on critical illness concepts. The authors are encouraged to clearly specify the scope of the review and refine the aim accordingly.

Response:

 The scope of the review and its aim have been refined. The intention is to address both concepts regarding critical illness as well as management. The revised aim is now more explicit and reads: *“The aim of this scoping review is to identify, synthesise, and map the concepts that underpin critical illness and the management of critically ill adult patients across the stages of the care pathway.”*

2. Comment:

 The authors are encouraged to clarify whether the review intends to comprehensively address management across all three phases or focus on specific aspects within each stage. Narrowing or clearly defining the scope of management within these phases would improve the clarity and feasibility of the objective.

Response:

 Our intention is to map a identified concepts across the care pathway, and whether these  pertain to patients, family members, healthcare professionals, and organisational or operational processes. The revised objective now reads: *“To identify the populations, clinical contexts, and stages of the care pathway (e.g. pre‑ICU, ICU, recovery) in which these concepts have been studied, including concepts relating to patient‑level management, family involvement, healthcare professional practice, and organisational or operational processes.”*

3. Comment:

 The population is described as adults; however, the inclusion criteria specify participants aged 16 years and above.  The authors should provide a clear justification for including individuals aged 16–17 years in the adult population. 

Response:

 The inclusion of individuals aged 16–17 years reflects that admission practices in some adult ICUs include patients within this age range. Using 16 years as the lower age threshold ensures maximum inclusiveness of potentially eligible search results based on the local practice of the ICU involved in the source study. An additional sentence has been added to clarify this rationale: *“This reflects admission age thresholds commonly used in adult critical care research and service provision.”*

4. Comment:

 The section describing the types of studies should clearly specify the time frame for the included studies. The authors are encouraged to mention the range of publication years considered in the review. 

Response:

 The timeframe for included studies is now explicitly stated within the *Types of studies* section. Studies of any publication year will be eligible, as the databases will be searched from inception to the date of the search. The following sentence has been added to the manuscript: *“Studies of any publication year will be eligible, as databases will be searched from inception to the date of the search.”*

# Reviewer report: Reviewer response for version 1

**DOI:** 10.3310/nihropenres.15386.r39399

**Published:** 2026-02-25

**Authors:** Lisa Salisbury

**Affiliations:** 1 Queen Margaret University, Musselburgh, Scotland, UK

This protocol proposes is a novel approach to gaining further understanding of the management of critically ill patients which is an important area of work as critical illness and recovery are complex and there are still large variations in practice and support individuals receive after critical illness.

 The protocol for this review is clearly reported and justified with detailed descriptions and explanations of steps that will be undertaken.

 In the plain English summary the term 'medical concepts' is used. This is the only place this term is used as other places it is 'clinical concepts' or just 'concepts'. Using the word 'medical' in this context may lead people to assume this is just concepts related to doctors rather then the multi-disciplinary team. Perhaps this could be reviewed and if 'medical' is the preferred term by the PFAG group perhaps the term rehabilitation could be included so it is 'medical and rehabilitation concepts' which represents the multi-disciplinary management of people after critical illness.

 In the Patient and Public Involvement section there is a full stop missing after (PFAG).

Is the study design appropriate for the research question?

Yes

Is the rationale for, and objectives of, the study clearly described?

Yes

Are sufficient details of the methods provided to allow replication by others?

Yes

Are the datasets clearly presented in a useable and accessible format?

Not applicable

Reviewer Expertise:

Evaluation of rehabilitation interventions after critical illness; Experiences of individuals after critical illness.

I confirm that I have read this submission and believe that I have an appropriate level of expertise to confirm that it is of an acceptable scientific standard.

# Response

**Published:** 2026-03-06

**Authors:** Kirsty Jerrard

**Affiliations:** Queen's University Belfast Wellcome-Wolfson Institute for Experimental Medicine, Belfast, Northern Ireland, UK

We thank the reviewer for taking the time to review our manuscript and for their helpful feedback. Our responses to the two comments are outlined below.

1. Comment:

 In the plain English summary the term 'medical concepts' is used. This is the only place this term is used as other places it is 'clinical concepts' or just 'concepts'. Using the word 'medical' in this context may lead people to assume this is just concepts related to doctors rather than the multi‑disciplinary team. Perhaps this could be reviewed and if 'medical' is the preferred term by the PFAG group perhaps the term rehabilitation could be included so it is 'medical and rehabilitation concepts' which represents the multi‑disciplinary management of people after critical illness.

Response:

 The plain English summary has been revised to ensure consistency with the main manuscript and to avoid implying a doctor‑specific focus. The term “medical concepts” has been removed and replaced with the broader phrase “key concepts” to reflect the multi‑disciplinary nature of critical illness care. The suggestion to use “medical and rehabilitation concepts” was considered; however, this phrasing introduces additional technical terms and was therefore not used in order to preserve plain‑language accessibility.

2. Comment:

 In the Patient and Public Involvement section there is a full stop missing after (PFAG).

Response:

 The punctuation has been corrected, and a full stop has been added after “(PFAG).”
